# Sympathetic Nerves and Innate Immune System in the Spleen: Implications of Impairment in HIV-1 and Relevant Models

**DOI:** 10.3390/cells11040673

**Published:** 2022-02-15

**Authors:** Denise L. Bellinger, Dianne Lorton

**Affiliations:** Department of Human Anatomy and Pathology, School of Medicine, Loma Linda University, Loma Linda, CA 92350, USA; ddifrancisco1@cox.net

**Keywords:** neural–immune interaction, splenic reorganization viral proteins, Tat, viral load and replication, chemokine receptors, type-1 interferons, M1 and M2 macrophages, blood-derived anti-inflammatory macrophages

## Abstract

The immune and sympathetic nervous systems are major targets of human, murine and simian immunodeficiency viruses (HIV-1, MAIDS, and SIV, respectively). The spleen is a major reservoir for these retroviruses, providing a sanctuary for persistent infection of myeloid cells in the white and red pulps. This is despite the fact that circulating HIV-1 levels remain undetectable in infected patients receiving combined antiretroviral therapy. These viruses sequester in immune organs, preventing effective cures. The spleen remains understudied in its role in HIV-1 pathogenesis, despite it hosting a quarter of the body’s lymphocytes and diverse macrophage populations targeted by HIV-1. HIV-1 infection reduces the white pulp, and induces perivascular hyalinization, vascular dysfunction, tissue infarction, and chronic inflammation characterized by activated epithelial-like macrophages. LP-BM5, the retrovirus that induces MAIDS, is a well-established model of AIDS. Immune pathology in MAIDs is similar to SIV and HIV-1 infection. As in SIV and HIV, MAIDS markedly changes splenic architecture, and causes sympathetic dysfunction, contributing to inflammation and immune dysfunction. In MAIDs, SIV, and HIV, the viruses commandeer splenic macrophages for their replication, and shift macrophages to an M2 phenotype. Additionally, in plasmacytoid dendritic cells, HIV-1 blocks sympathetic augmentation of interferon-β (IFN-β) transcription, which promotes viral replication. Here, we review viral–sympathetic interactions in innate immunity and pathophysiology in the spleen in HIV-1 and relevant models. The situation remains that research in this area is still sparse and original hypotheses proposed largely remain unanswered.

## 1. Introduction

As of 2017, an estimated 38 million people in the world are living with human immunodeficiency virus-1 (HIV-1), the pathogen in acquired immunodeficiency syndrome (AIDS) [1,2]. New infections in 2017 account for 1.8 million new cases. As of 2015, AIDS treatment was estimated at USD 24 billion annually in the United States, but sadly funding for treatment fell short by USD 7 billion [2,3,4]. There are three stages of HIV infection [2]. Acute HIV infection develops within two to four weeks post-infection, and manifests as flu-like symptoms (e.g., fever, headache, rash). In this stage, HIV-1 becomes systemic with high circulating viral titers, and assaults the immune system. During chronic disease, HIV-1 continues to replicate in targeted organs, but circulating levels are low, and the disease may be asymptomatic. AIDS is the final and most severe stage of disease due to a markedly compromised immune system. AIDS increases vulnerability to opportunistic infections due to low CD4^+^ T-helper (Th) cell counts, high viral load and immune dysfunction. Despite the large number of people living with HIV-1, life span is increasing in patients treated with combination antiretroviral therapies (cART) [2]. In these patients, HIV-1-related deaths result from cancers, cardiovascular disease, and HIV-1-associated neurocognitive disorder (HAND) [2]. cART reduces circulating viral load to undetectable levels. Still, HIV-associated morbidity remains, consistent with a latent viral reservoir, persistent immune dysfunction, and fibrosis in immune organs [4]. Fibrosis plays a major role in mediating chronic inflammation and abrogating the development of a robust immune response against the virus [5]. HIV-induced fibrosis disrupts the stromal network of the spleen, impairing immune cell trafficking and function. Stromal changes and immune deficiency persist during cART. AIDS in humans, monkeys and mice infect macrophages and CD4^+^ T cells, including those that reside in secondary lymphoid organs, such as lymph nodes and spleen [6,7]. The spleen is superimposed within the circulatory system, and plays a key role in viral persistence [8]. 

The sympathetic nervous system (SNS) innervates secondary lymphoid organs, and is activated by a variety of stressors, including viral infection [9,10]. Sympathetic dysfunction in HIV-1 infection exists as a continuum of early hyper-to-hypo-adrenergic function with disease progression [11]. Sympathetic activity is linked to prognostic disease indicators of HIV pathogenesis [12,13,14,15,16], as well as in animal models [17,18]. Further, drugs impacting the SNS in HIV [19,20], and simian immunodeficiency virus (SIV) [21,22] alter viral load and immune function. In people who become HIV-positive, high sympathetic reactivity is associated with augmented plasma viral loads, and importantly, impaired virologic responses to cART [14,16]. Individual differences in sympathetic reactivity, and one’s ability to cope with stressors, significantly contribute to the relationship between stress or temperament-related risk factors and virologic/immunologic indicators of HIV-1 pathogenesis [15,16]. These findings are consistent with studies in monkeys reporting enhanced replication of SIV in infected cells adjacent to catecholamine-containing varicosities of sympathetic nerves in primate lymph nodes [22]. Collectively, historical, anatomical, physiological and pharmacological studies support that the SNS can mediate biobehavioral influences on the pathogenesis of SIV and HIV-1. 

The SNS regulates innate and adaptive immune responses against HIV-1 infection through its innervation of lymphoid organs and blood vessels. Sympathetic nerves innervate immune cells and related vasculature in lymph nodes and spleen [23]. These nerves secrete norepinephrine (NE) as their main neurotransmitter, as they course along smooth muscle, endothelial cells and microglial-like pericytes in blood vessels where they regulate their activity [23]. These nerves regulate blood flow through the red and white pulps as they travel along the blood vessels that supply the spleen. They then exit the vasculature, entering into the white pulp and to a lesser extent, the red pulp, where they regulate immune cell functions and responses to infection [23,24,25]. 

With SIV, simian-human immunodeficiency virus (SHIV) or HIV-1 infection, splenic lymphocytes undergo lytic processes, whereas macrophages acquire a reservoir [26,27]. Splenic macrophages are important contributors to SIV and HIV infection in the spleen, driving morphologic changes through the specific loss of normal macrophage subsets that mediate splenic organization and impair systemic immune functions [24,28]. Therefore, in HIV-1 and animal models, the spleen undergoes remarkable pathologic changes that impair neural–immune regulation of innate and adaptive immunity. Early research in murine acquired immune deficiency syndrome (MAIDS) supports an early negative effect of viral infection on the SNS. Kelly et al. [29] report severe sympathetic neuropathy in the spleens of mice with MAIDS two weeks after infection. Later, Sloan and colleagues [30] extend this finding in axillary lymph nodes in SIV-1 infection, an effect associated with reduced nerve growth factor, a required factor for nerve integrity. These findings indicate that SIV-1 infection destroys nerves that normally regulate immune responses to pathogens, subjecting infected patients to the deleterious effects of opportunistic infections. Sloan et al. [30] was the first to identify a potential mechanism of sympathetic nerve loss in the infected spleen with SIV-1 infection.

More recently, sympathetic hyperactivity, systemic vascular dysfunction and neuropathy have been reported in cART-treated patients with well-controlled HIV-1 infection [11,31]. This finding is associated with an elevation of the proinflammatory cytokine, IL-6, and occurs in the absence of changes in tumor necrosis factor (TNF)-α, pain, or anxiety [11,32]. In an HIV-1 transgenic rat model, HIV-1 induces apoptosis in vascular endothelial cells and splenocytes [24]. Inflammatory cytokines, loss of immune target cells, and psychosocial stress are all detrimental to sympathetic nerve terminals. These factors are likely contributors to the loss of sympathetic nerves in spleens from individuals with HIV-1 infection and animal models of HIV-1 [29,30,31,32,33,34].

Here, we review the complex interaction between the SNS and antiviral innate immunity in HIV-1 infection and animal models of HIV-1. One of our goals is to revisit the current literature to better understand sympathetic nerve loss reported in the spleen in MAIDS [29] over two decades ago, and related newer research. The marked early sympathectomy after LP-BM5 inoculation has largely gone unnoticed by the scientific community, possibly because the findings were difficult to explain and its implications were lacking at the time of publication. However, a more recent clinical report in HIV-1, as well as a murine study with a related retrovirus, suggests that sympathetic nerve loss may be a consistent finding during acute disease with HIV-1 infection or other retroviruses, with consequences for severity of infection [33,34]. We revisit this research and evaluate the existing literature to advance a unifying hypothesis regarding the mechanism(s) that explain(s) the striking findings reported in Kelley et al. [29,32], and their importance for understanding HIV pathology based on new findings that were sorely lacking at the time that this research was carried out. Here, we (1) focus on the impact of HIV-1-induced pathology in the spleen, including sympathetic innervation, and their consequences for innate immunity; (2) describe animal models for HIV-1 (specifically, murine and simian models); (3) discuss how MAIDS infection causes pathology in the spleen; (4) evaluate the impact HIV-1 and related experimental viruses on innate immune functions; and (5) discuss the impact that the “loss-of-functions” of splenic sympathetic nerves and the immune system have on their ability to defend against opportunistic infections. While current research has advanced our understanding of sympathetic regulation in the pathophysiology of HIV-1 and relevant animal models of HIV-1, many important questions remain unanswered.

## 2. MAIDS and Simian AIDs (SAIDS) as Models for HIV-1

### 2.1. MAIDS Model

To understand the etiology/pathophysiology, and to identify treatments for HIV-1, several animal models have been developed. In this review, we primarily focus on two models, MAIDS and SAIDS. MAIDS was one of the earliest models used to study HIV-1 infection. LP-BM5 is a defective murine leukemia virus that induces MAIDS in genetically susceptible mice. MAIDS is characterized by early dysregulated activation of the immune system and death within 4–6 months [35,36,37,38,39,40,41,42]. LP-BM5, a murine retrovirus isolate, induces a murine immunodeficiency syndrome in genetically susceptible mice, such as the C57BL/6 (B6) strain. This highly pathogenic isolate contains a mixture of murine leukemia retroviruses. These include ecotropic, recombinant mink cell cytopathic focus-inducing, and replication-negative/defective viruses, with the defective genome serving as the proximal etiological agent of disease [35,36,40,41]. Many of the described disease features of the LP-BM5-induced syndrome are similar to those occurring in human AIDS, and are hence referred to as murine AIDS or MAIDS. Similarities include hypergammaglobulinemia, splenomegaly, lymphadenopathy, dependence on CD4^+^ T cells for initiation of disease, severely dampened T and B cell responses, increased susceptibility to infection and death when exposed to normally nonpathogenic microorganisms, and the development of terminal B cell lymphomas [35,36,40,41].

**Figure 1:**
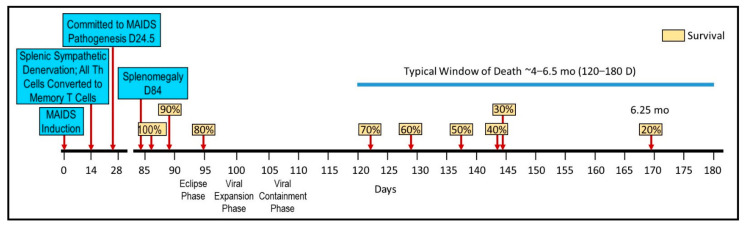
The timeline for significant early pathologic events after murine acquired immunodeficiency (MAIDS) induction in C57Bl/6 mice (blue boxes), and the estimated percent survival rates (yellow boxes) across time are expressed in days and/or months based on Umemura et al., 2002 [42]. After MAIDS induction, profound sympathetic nerve loss in the spleen is evident fourteen days after disease induction. A commitment to develop MAIDS occurs by 24.5 days, and splenomegaly is evident by 84 days after inoculation with LP-BM5. Survival rates across time are indicated in yellow boxes, and the typical window of death from MAIDS (4–6.5 months (mo) or 120–180 days (D)) is indicated by the horizontal blue bar. This timeline is constructed in part from Umemura et al., 2002 [42]. Th, T-helper.

Figure 1 illustrates the timeline for early pathologic events in MAIDS after onset. These events include disease-induced sympathectomy, as an early event, and the later development of splenomegaly, as well as the estimated percent survival rates across time after the induction of MAIDS. The loss of sympathetic nerves occurs ~14 days after disease induction, whereas splenomegaly is apparent by day 84 after inoculation with LP-BM5 [42]. In a number of important ways, the pathogenesis of MAIDS is similar to AIDS. These include: (1) requires pathogenic Th cells to initiate disease and progression; (2) enlarged spleen and lymph nodes; (3) elevated circulating immunoglobulin levels; (4) changes in T cell differentiation; (5) the subsequent loss of functional Th cells and severely depressed T and B cell-mediated immune responses; (6) altered cytokine production, (7) greater susceptibility to infections and mortality from exposure to normally nonpathogenic microbes; (8) susceptibility to opportunistic neoplasms (i.e., B cell lymphoma) [43,44,45]; and (9) oxidative stress [38]. In contrast to HIV-1, infection with LP-BM5 retrovirus targets highly immunosuppressive myeloid suppressor cells [36], which severely dampen both T and B cell functions, whereas HIV-1 directly infects CD4 Th cells. Regardless of this difference between AIDS and MAIDS, the LP-BM5 retrovirus is widely used as a model to investigate HIV-induced changes in immune functions [35,36,38,39,40,46,47]. Finally, the MAIDS model has significantly and cost-effectively advanced our understanding of AIDS pathogenesis, cofactor involvement, and particularly, testing for novel treatments for HIV-1 and human AIDS [36,37].

Neither HIV-1 nor LP-BM5 infect neurons. Sympathetic nerve loss in the spleen occurs secondary to the inflammation that ensues with LP-BM5 infection. Additionally, HIV-1 Tat contributes to sympathetic nerve loss, a protein that is not secreted by LP-BM5 [48]. C57Bl/6 mice infected with defective LP-BM5 retrovirus [35,49] develop a fatal MAIDS characterized by lymphoproliferation and profound immunodeficiency [39].

Still, MAIDS differs from HIV-1 [29]. In MAIDS, lymphoproliferation and marked immunosuppression begin with virtually no latency period. Virtually no immune function is detectable by four weeks post infection, whereas HIV-1 infects Th cells, and LP-BM5 infects macrophages and B and Th cells. Polyclonally activated B cells actively secrete IgM or IgG [50,51]. For a brief period, immune responsiveness increases, but is quickly followed by long-lasting profound cellular and humoral immune suppression. In MAIDS, the mechanism of suppressed B cell functions may be unique. B cells are largely polyclonally activated, antibody-secreting plasmablasts. These cells employ a T cell-dependent pathway of B lymphocyte activation. 

Infected mice died 4–6 months after viral inoculation, and displayed massive lymphadenopathy. While MAIDS shares many common features with HIV infection, the fate of Th cells in MAIDS starkly differs from HIV infection [39,52,53]. The cell surface protein that mediates apoptosis, CD95 (APO-1/Fas) [52], is increased in CD4^+^ and CD8^+^ T cell subsets in HIV-1 infection [54]. CD95 mediates apoptosis-induced depletion of CD4^+^ T cell in AIDS [55,56]. Similar to human AIDS, Fas expression is also increased in Th and B cells in the lymph nodes and spleen of mice with MAIDS [57]. However, in contrast to human AIDS, mice with MAIDS are characterized by lymphoproliferation of CD4^+^ T and B cells due to a faulty Fas/Fas ligand (Fas L) system as MAIDS progresses [57]. Despite increased functional Fas and Fas L expression in MAIDS, CD4^+^ T cell numbers rise as disease progresses [57]. The Fas/Fas L system is not involved in the progression of MAIDS, but is a participant in both SIV and HIV-1 pathology [58]. In MAIDS, there is no latency period for lymphoproliferation and immunosuppression, and the immune system is severely suppressed. By 4 weeks after infection, virtually no immune function is detectable. Despite the profound immunosuppression in MAIDS, these mice most commonly succumb to respiratory failure due to the enlargement of mediastinal lymph nodes, not from opportunistic infections. This difference from the cause of death in AIDS may be attributed to the more sterile housing conditions that minimize exposure to opportunistic infections in MAIDS. 

Despite divergent clinical progression in AIDS versus LP-BM5-induced lymphoproliferation, the early similarities in MAIDS and AIDS, as well as the common retroviral etiology, and available preclinical research support MAIDS as a useful model for enhancing our understanding of retrovirus-mediated pathology in HIV-1. Therefore, the MAIDS model is an established preclinical model for HIV-1. The similarities in immunopathology of MAIDS and human AIDS, and the continued use of MAIDS for preclinical testing of promising therapeutics, strongly supports its use for investigating immune-mediated pathogenesis and treatment of AIDS. The MAIDS model has substantially informed both the medical strategies for treating AIDS, and the pathophysiology of AIDS [39,59,60,61,62]. 

### 2.2. SAIDS

SIV is a species of retrovirus that causes persistent infection in many African non-human primates [62]. The SIV model for HIV-1 has taken many decades to evolve, partly due to SIV’s natural infection and lower virulence of SIV in certain African species, despite high viral loads (e.g., sooty mangabeys and African green monkeys). Many models of pathogenic SIV infection have been developed in macaque species (rhesus, pigtail, cynomolgus, etc.), with several strains of simian virus (e.g., SIVmac (macaques), SIVsm (sooty mangabey, an Old-World monkey), as well as simian-human immunodeficiency viruses (SHIVs) [63]. Typically, SIV causes persistent chronic infection, but generally not acute disease. SIV progresses to SAID within months to years, depending on the strain of SIV. Currently, SAIDS in macaques is considered the best available animal model for studying the pathogenesis of AIDS, and for developing antiretroviral therapeutics to treat AIDS [64] in the SIV model with persistent infection that leads to immunodeficiency, opportunistic infections, and death [63]. 

The advantage of the SIV model over rodent models is their development of pathogenic SIV and SAIDS that is similar in many ways to human AIDS [61,63]. The SIV model permits researchers to carry out experimental immunological interventions/manipulations that are inappropriate, too risky, or that cannot be conducted in people. Additionally, using recombinant SHIV or HIV-1 derivatives, macaques are the most commonly used and widely accepted model for HIV/AIDS, particularly for central nervous system (CNS) pathology in macaques, which are similar to HIV-1 in humans [65,66,67]. 

## 3. Splenic Sympathetic Nerves Influence HIV-1 Cell Entry and Replication in Acute Disease

The spleen, and also lymph nodes, play a role in innate immunity to viral penetration, in addition to its role in adaptive immunity. The spleen has two main histologic compartments, the red and white pulps [68]. The red pulp mainly filters the blood for pathogens and removes old senescent red blood cells. The splenic white pulp houses the majority of the bodies’ lymphocytes, and is a site where innate immune cells present viral antigens to T cells to initiate adaptive immunity. Sympathetic noradrenergic fibers enter the spleen with the vasculature, and course along the trabeculae and branching vasculature. These nerves are mainly distributed in the white pulp by coursing along the central arterioles and in the adjacent periarteriolar lymphatic sheath. These nerve fibers branch from a dense plexus around the central arteriole and travel into the parenchyma, where they end up among fields of lymphocytes and other cell types. Noradrenergic innervation is generally directed into zones of T lymphocytes and plasma B cells rather than into follicular regions that house B lymphocytes. Innate immune cells lack β_1_-adrenergic receptors (ARs), but express β_2_-, α_1_-, and α_2_-AR subtypes, while immune cells that participate in adaptive immunity predominantly express β_2_-ARs [68,69]. The SNS largely regulates innate and adaptive immunity with the goal of maintaining homeostasis via these receptors. 

**Figure 2:**
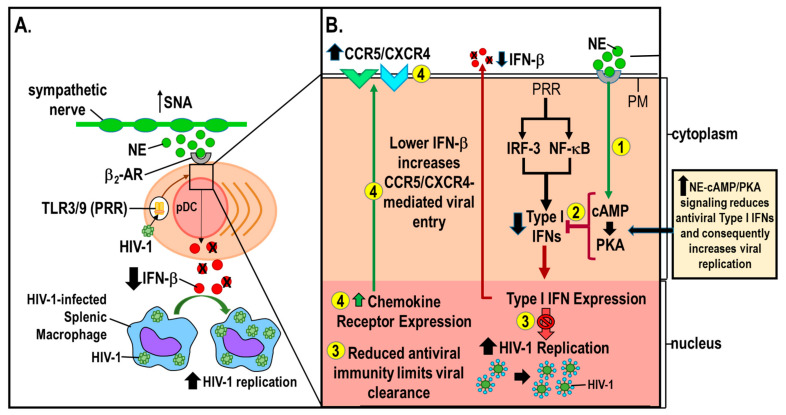
The influence of sympathetic nerves in establishing human immunodeficiency virus-1 (HIV-1) infection. (**A**) HIV-1 can infect cells of the immune system (e.g., plasma dendritic cells (pDC); cytoplasm tan; nucleus, pink) via binding to Toll-like receptors (TLR)3/9, and activate their intracellular signaling pathway that induces the production of Type I interferons (IFNs), particularly IFN-β. HIV-1 infection increases sympathetic nerve activity (SNA) stimulating greater norepinephrine (green circles) transmitter release from sympathetic nerves that activate signal transduction (black box) in splenic plasmacytoid dendritic cells. Norepinephrine (NE) binding to β_2_-adrenergic receptors (β_2_-ARs) (gray semicircle) activates its intracellular signaling pathway (black box and arrow) that suppresses (black Xs) IFN-β secretion (red circles). Reduced IFN-β secretion by pDCs diminishes the suppressive effect of IFN-β on HIV-1 replication in HIV-1-infected splenic macrophages (black upward arrow). (**B**) The modulatory influence of β_2_-AR activation by NE on the intracellular signal transduction of virally-activated pattern recognition receptors (PRR)-induced stimulation of type I IFNs intracellular signaling pathway is illustrated. During acute HIV-1 infection, increased NE (green circles) ligation of β_2_-AR stimulates cAMP-induced protein kinase A (PKA) (yellow circle 1), which inhibits the cellular production of type I IFNs and secretion of IFN-β (yellow circle 2) and suppressed type I IFN disinhibits anti-viral replication in the nucleus (red arrow with 🚫), limits antiviral immunity and viral clearance (yellow circle 3), and increases production and cell surface expression CCR5 and CXCR4 (green arrow), receptors used by HIV-1 to infect immune cells. Reduced secretion of IFN-β (black downward arrow) promotes, creates a permissive microenvironment for the HIV-1 infection in secondary immune organs by reducing innate antiviral immunity. During chronic infection in a rodent model of HIV-1 (when infection is well-established), sympathetic innervation of the spleen is lost (in part by chronic inflammation). The destruction of these nerves removes a major neural regulator of cellular and adaptive immunity, which contributes to an increase in the risk of opportunistic infection. Double black lines, plasma membrane PM; IRF-3, interferon regulatory factor 3—a transcription regulator of cellular responses essential for innate immunity; NF-κB, nuclear factor kappa B; C-C chemokine receptor type 5, CCR5; C-X-C chemokine receptor type 4 (CXCR-4).

HIV-infected individuals with constitutively high activity of the autonomic nervous system before antiretroviral therapy experienced lower suppression of plasma viral load and poorer 3-month CD4^+^ T cell recovery [14,70]. This finding is consistent with physiological concentrations of catecholamines (10^−8^ to 10^−5^ M) significantly augmenting HIV-1 replication in vitro [71], a response mediated by the β_2_-AR-cAMP-protein kinase A (PKA) signaling pathway [70] (see Figure 2). These findings are also consistent with psychosocial stress negatively impacting clinical outcomes [9,10]. Similarly, combined in vitro treatment with a PKA activator alone or synergized with a histone deacetylase inhibitor (an epigenetic modifier of PKA) reactivated latent HIV-1 in infected cells [71,72]. These findings support that sympathectomy in the spleen, via lost PKA signaling, may lower circulating viral load and promote viral latency.

Early research investigating lymph nodes from rhesus macaques infected with SIV reveals a key role of sympathetic neurons in regulating viral replication in secondary lymphoid tissue. Sloan and coworkers report greater SIV replication adjacent to sympathetic varicosities, a site of catecholamines release in the lymph node parenchyma [22]. Similarly, NE, via β_2_-ARs and other activators of cAMP/PKA, enhance replication of HIV-1 [70]. A subsequent study uncovered a substantial degree of behaviorally induced plasticity in sympathetic innervation of lymphoid organs. Macaques subjected to experimentally imposed social stress show elevated density of catecholaminergic varicosities in the lymph node parenchyma [20]. Stress also enhances SIV replication by 3.9-fold, an effect attributed specifically to a greater density of catecholaminergic varicosities. 

A more recent study in adult rhesus macaques finds that a β-AR antagonist enhances the density of sympathetic innervation, an effect associated with higher SIV gene expression in lymph nodes [22]. This is further supported by findings that HIV-1-positive patients that had constitutively high autonomic activity prior to cART had less reduction in plasma viral load and less CD4^+^ T cell recovery during the 3–11 months of cART therapy. This same study finds that NE elevated the replication of two different strains of HIV-1 in vitro. The increase in viral replication is accompanied by an upregulation of chemokine receptors (CCR5 and CXCR4) and augmented viral gene replication [14]. These findings suggest that sympathetic nerves are protect against the damaging effects of oxidative stress from free radicals, and provide an experimental link between the modulation of the SNS and viral replication. The graphic abstract for this paper illustrates a modified framework by Cole et al. [14] that integrates existing findings from (1) clinical studies relating sympathetic activity to indicators of HIV-1 pathogenesis [14], (2) in vitro assessment of catecholaminergic effects on HIV-1 replication [14,70,73], and (3) in vivo experimental findings in lymphoid tissues in the SIV model [21,22]. The model proposes sympathetic activity enhances viral replication by suppressing the activity of Type I interferons (IFN-β) [74]. 

Type I interferons produced and secreted by infected cells exert several major functions: (1) induce an intrinsic intracellular antimicrobial state within the infected cell, and in neighboring cells to limit viral spread; (2) modulate innate immune responses in a balanced way that facilitates antigen presentation and natural killer (NK) cell activation; and (3) induce activation of an adaptive immune response [75]. Regarding NK cells, HIV-1 infection alters NK cell homeostasis, downregulates cell surface CD56 expression and alters the expression of other cell surface markers [76,77]. HIV-1 also interferes with their antiviral effector functions, and, under conditions of high chronic HIV-1 viremia, markedly interferes with NK cell functions that regulate the crosstalk between innate and antiviral adaptive responses early in disease. HIV-1 infection redistributes NK cell subsets and expands anergic CD56^neg^ NK cells that express an aberrant repertoire of activating and inhibitory receptors [76,77]. Despite these virally-induced aberrations in NK cells, specific haplotypes for NK cell receptors, and NK cell antibody-dependent cell cytotoxicity can control HIV-1 infection. During chronic HIV-1 infection, circulating NK cell numbers rebound. Additionally, cART can restore both NK cell phenotype and functions to normal levels in HIV-1-infected patients [76,77]. Increased NK cell activity results from greater cytotoxic capacity, IFN-γ and chemokine production (CCL3, CCL4, and CCL5), and is effective in resisting HIV infection, lowering viremia-controlling infection, and delaying progression to AIDS [77].

In a longitudinal study in pigtail macaques, SIV infection profoundly alters gene expression in the spleen [78]. They find an upregulation of (1) interferon and interferon-stimulated genes, (2) M2 macrophage polarizing genes, and (3) monocyte/macrophage chemokines and chemokine receptor signaling. The monocyte/macrophage chemoattractant, CCL8 (aka. monocyte chemotactic protein-2) is increased (~200-fold) compared with uninfected macaques. This is the most differentially regulated transcript during SIV infection. The methods used by Williams et al. [78] did not allow them to identify the specific cell source of interferon production, the subtype of M2 polarizing macrophages, or the cells that upregulated the gene expression of chemokine and chemokine receptors. Splenic macrophages significantly influence SIV replication and sufficiently maintain viral production in the absence of T cells. Based on this study, SIV destroys the splenic macrophages, and recruits inflammatory peripherally derived blood mononuclear cells that promote an anti-inflammatory microenvironment in the spleen. These findings indicate that SIV pathogenesis is largely driven by alterations in and infection of macrophage subsets. This process is an early event that continues through chronic disease. Although, they report that initially Type I interferon production in the spleen is elevated, its production is markedly suppressed in the late stage of SIV infection [78].

Reduced Type I interferons increase viral replication through multiple mechanisms. They include impaired resistance to viral gene expression by inhibiting the interferon-mediated antiviral state [79], and enhancing the cellular vulnerability to infection. The increase in cellular vulnerability occurs by disinhibiting the expression of viral co-receptors, CCR5 and CXCR4. The latter mechanism occurs under physiologic conditions [80,81,82,83], but not in artificially stimulated cells [84]. In conjunction with immune activation (e.g., via proinflammatory cytokines or ligation of the T cell receptor), these factors facilitate viral replication, and thereby, accelerate disease pathogenesis.

HIV-1 infects pDC, macrophages and CD4^+^ T cells in the splenic white pulp, where HIV-1 replicates and then establishes a reservoir of latent virus within these cells during chronic disease [85,86,87]. Neural HIV pathology also includes inflammation-mediated destruction of sympathetic nerves that normally regulate the immune functions of these three cell types [11,31,32]. As a result, the spleen is a critical site involved in the progression of HIV-1 infection towards clinical immune deficiency and greater susceptibility of infected individuals to opportunistic disease [88,89,90,91]. Heightened activity of the SNS is consistently linked to driving the pathogenesis of HIV-1, increased vulnerability to opportunistic infections, neurocognitive decline, and cardiovascular complications [92,93,94,95]. 

Co-expression of CCR5 and CXCR4 in monocyte-derived dendritic cells promote inflammation and serve as co-receptors for HIV-1 entry into host cells. Sympathetic signaling to dendritic cells can, through the release of NE and dopamine, affect the expression of both of these chemokine receptors [96]. First, dopamine, co-released with NE from sympathetic nerves, can affect CCR5 expression on innate immune cells [97]. Dopamine activation of dopamine receptor (DR)D1 and DRD4 expressed on innate immune cells regulates nuclear transcription factors that bind to sites in the CCR5 promoter [97]. Ligation of these receptors suppresses CCR5 transcription. Conversely, dopamine also regulates the expression of the HIV-1 protein, Tat, which enhances cell surface expression of CCR5. Secondly, NE binding to β_2_-ARs increases cAMP, which exerts a proviral effect in HIV-1 infection by externalizing CXCR4 on the cell membrane of lymphocytes [72,98] (see Figure 2). Similarly, methamphetamine, which increases catecholamine uptake and release in catecholaminergic neurons in the CNS, enhances CXCR4 and CCR5 to promote viral entry in monocyte dendritic cells [98]. Upregulated cell surface expression of the viral co-receptors, CXCR4 and CCR5 [14,72] by cellular transcription factors, enhances transcription of HIV-1 genes [14]. Additionally, catecholamines released by sympathetic nerves also suppress Type I interferon responses to HIV-1 infection [73]. Thus, sympathetic nerves have multiple ways to influence HIV-1 viral replication in acute disease; these effects are lost as disease progresses and sympathetic nerves degenerate.

All in all, current research is consistent with the SNS as a mediator of biobehavioral influences on HIV-1 pathogenesis. In cultured PBMCs, β_2_-AR stimulates a rise in cAMP, suppresses antiviral immunity, increases the vulnerability to infection by upregulating CCR5 and CXCR4 expression, enhances viral gene expression in the host genome by targeting transcription factors for activation, and suppresses the type I interferon response (see Figure 2). Variations in SNS activity may mediate the relationships between psychosocial risk factors and HIV-1 replication [12,15]. Similarly, research in the SIV model identifies a key role for stress-induced remodeling of sympathetic innervation in lymphoid tissues as an important influence on viral replication [21,22]. 

## 4. HIV-1 Establishes Cellular Reservoirs and Induces Splenocyte Dysfunction 

### 4.1. HIV-1-Induced Pathogenesis in the Spleen and Establishing the Viral Reservoir

In the spleen, lymphocytes and macrophages are early targets of HIV-1 infection. The white pulps and germinal follicles become depleted of T and B lymphocytes, respectively. This disrupts splenic morphology and drives suppression of cellular and humoral immunity [72]. HIV-1 also infects distinct subsets of macrophages that reside in the red and white pulp, each with diverse functions based on where they reside in the spleen [99,100]. The marginal zone delineates the interface between the red and white pulps. The inner most region of the white pulp contains the periarteriolar lymphatic sheath that surrounds the central arteriole. The periarteriolar lymphatic sheath is primarily a T cell zone. Dendritic cells and macrophages reside in the marginal zone of the white pulp, where they trap blood-borne antigens for antigen processing and presentation [101]. These cells present viral antigens to Th cells to initiate an antiviral immune response [102]. In the spleen, the main producers of IFN-β are pDCs, the primary immune cell involved in innate immunity against viral infections [103]. They are “powerhouse” innate immune cells, essential for producing antigen-specific, high-affinity plasma and memory B cells [104,105,106]. Tingible body (stainable) macrophages detect, engulf and phagocytize many apoptotic HIV-1-infected T cells in the marginal zone, and then migrate to germinal centers for antigen presentation to follicular B cells [101]. Red pulp macrophages have three important functions: (1) phagocytose erythrocytes, (2) scavenge hemoglobin and haptoglobin, and (3) recycle and store iron. Thus, splenic macrophage subclasses display unique functions in detecting HIV-1 and HIV-1-infected cells, depending on where they reside. They differentially express cell markers and reside in distinct compartments that change after HIV-1 infection, and as the innate immune response ensues [101]. 

**Figure 3:**
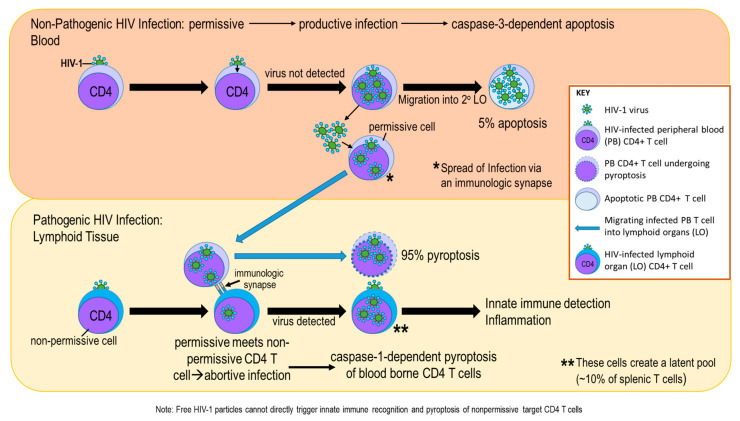
Tissue-specific programmed cell death in HIV-1-infected CD4 T-helper (Th) cells: determines HIV pathogenesis. Upper panel: HIV-1 virions productively infect and lyse peripheral blood CD4^+^ T cells. This occurs via caspase-3-mediated apoptosis in less than 5% of all CD4 T cells, and without directly inducing the recognition of the innate immune system. This is a normal mechanism for removal of infected or cells damaged beyond repair. A small subset (~5%) of vulnerable cells become infected via pattern recognition receptors (PRRs). Infection drives apoptosis in these cells. These infected cells are “assassins” (blue arrow from upper to lower panel) responsible for CD4 T cell depletion in the spleen (shown in lower panel) by viral spread to uninfected Th cells. Lower panel: In contrast, HIV-1 viral particles induce pyroptosis in non-permissive CD4 Th cells in the spleen. These cells do not support growth or genetic replication of HIV-1, and therefore resist productive HIV-1 infection. Pyroptosis accounts for more than 95% of splenic CD4 Th cell death. This markedly induces inflammation. Thus, HIV-1 indirectly kills nonpermissive target CD4 T cells. HIV pathogenesis promotes CD4 T cells depletion and a state of chronic inflammation that facilitates HIV-1 progression to AIDS. These two mechanisms are not independent, but rather linked as a single pathogenic cascade (blue arrow). Once circulating infected CD4 T cells enter the spleen, they undergo pyroptotic cell death. See reference [108] for a more detailed account. PB, peripheral blood; LO, lymphoid organs; HIV, human immunodeficiency virus; black arrow, directional progression; blue arrow, migration into lymphoid organs.

As HIV-1 infection progresses, infected effector Th cells die and release HIV-1. Released HIV-1 then infects adjacent Th cells causing them to die. Virus-induced Th cell killing results in hyposplenia, as thymus-derived T cells that home to the spleen are unable to sufficiently keep pace with high HIV-1-induced T cell loss. In contrast, HIV-1-infected splenic macrophages are long-lived cells that become HIV-1 viral reservoirs. Additionally, the longer-lived memory T cells are preferentially infected with HIV-1, and serve as another viral reservoir [107]. The cellular debris from HIV-1-induced lytic cell death in infected Th cells is a potent initiator of inflammation. Macrophages also play a pivotal role in the inflammatory and early innate immune responses to the viral infection. Cell death of HIV-1-infected immune cells in the spleen occurs mainly by caspase-1-mediated pyroptosis (~95%), a highly inflammatory form of lytic programmed cell death (see Figure 3). In contrast, only about ~5% of infected Th cells undergo apoptosis, a form of programmed cell death that serves as a gatekeeper of host defense against invasion by certain pathogens. Proinflammatory signals trigger necroptosis in inflammatory macrophages infected with HIV-1 [108]. In contrast, pyroptosis uses an alternative intracellular pathway (i.e., caspase-1) to carry out cell death in T cells. Both of these alternative mechanisms of cell death release molecules that further drive inflammation in the HIV-1-infected spleen. 

### 4.2. Spleen Dysfunction in SIV and HIV-1 Infection

HIV-1 infection profoundly disrupts the organization, cellular constitution, and functions of the spleen. Pathologic changes include perivascular hyalinization, infarction, necrosis and granulomatous reactions [109]. The white pulp is diminished from loss of infected CD4^+^ T cells, and bystander damage. In addition to T cell loss and dysfunction, macrophages substantially contribute to splenic destruction and dysfunction. Infected macrophages promote CD4^+^ T cell death, but before their demise, T cells spread HIV-1 infection to other T cells. [110,111,112]. In contrast, B cell-derived germinal centers remain functional despite viral infiltration [113]. Virally infected T cells and resident macrophages become cellular reservoirs for HIV-1 in the spleen [108,114], and consequently, these cells lose their normal functions. 

HIV-1 induces the downregulation of functional markers in splenic macrophages that impairs their activation and their ability to filter the blood. This further promotes splenic pathology, and poor clinical outcomes, including opportunistic infections in HIV-1-positive individuals [115]. Histologic changes in the HIV-1-infected spleen (as well as SIV) include (1) markedly depleted CD4^+^ T cells from apoptotic cell death; (2) increased recruitment of virus-specific cytotoxic CD8^+^ T cells; (3) greater numbers of latently infected memory T cells [108,116,117,118]; and (4) development of a major latent viral reservoir in infected splenic macrophages. Infected macrophages become the major latent viral reservoir during chronic infection. This reservoir allows for viral persistence and continual damage to the spleen [115,119,120]. Pathology also includes the migration of blood monocyte-derived macrophages into the spleen. These cells differ from resident macrophages in that they drive inflammation and initiate a distinctive antiviral response to viral infection in the spleen [121,122,123]. 

Similarly, SIV infection profoundly drives a long-lasting shift in splenic macrophage populations from the normal M1 towards a monocyte-derived M2 phenotype [78]. In this shift, the number of red pulp M1 macrophages declines, and macrophages in the white pulp polarize toward an M2 microglial-like phenotype. Cell death of splenic M1 macrophage subpopulations is primarily responsible for the HIV-1-induced loss of CD68^+^ macrophages. In contrast, increased CD68^+^CD163^+^ M2 macrophages in the white pulp results from infiltration of infected monocyte-derived macrophages. These M2 macrophages markedly impair splenic function by suppressing the inflammatory and antiviral responses required to eliminate the SIV infection. With their infiltration, resident splenic CD68^+^ macrophages profoundly decline, including perivascular macrophages that co-express CD163. In the red pulp, apoptosis of microglial-like CD68^+^ macrophages markedly limits the potential for restoring this population in the spleen. The loss of resident CD68^+^ macrophages increases the risk of opportunistic infections due to the inability to mount an effective adaptive immune response. Loss of these cells also impairs the clearance of damaged erythrocytes and scavenging of iron in SIV infection [78]. 

**Figure 4:**
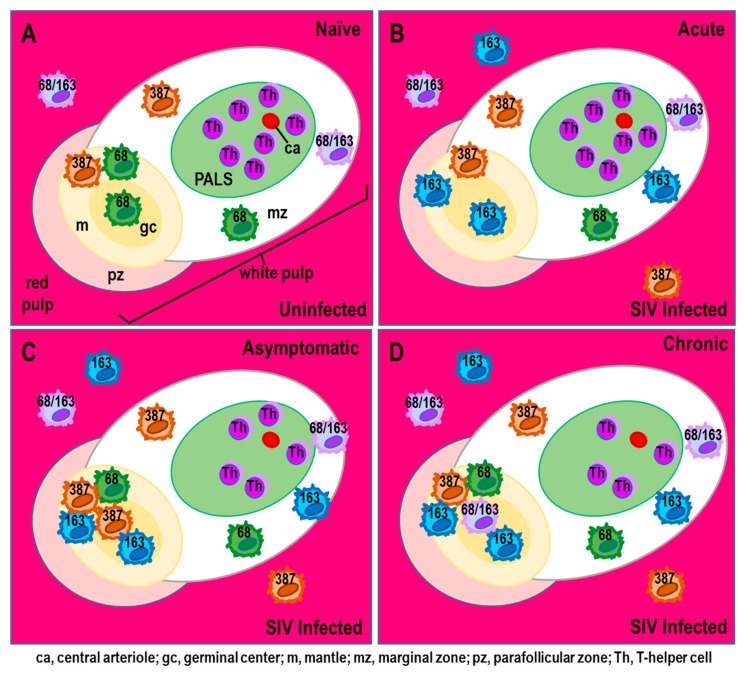
A schematic representation of changes in macrophage populations in the spleen in (**A**) uninfected controls (Naïve), (**B**) Acute, (**C**) Asymptomatic chronic, and (**D**) Chronic SIV infection based on the research of Williams et al. [78] in the American Journal of Pathology. Normal and altered splenic macrophage populations are illustrated diagrammatically at different stages of SIV infection. (**A**) In the germinal centers in uninfected spleens (homeostasis), the dominant macrophage subset is CD68^+^ cells (green). In contrast, the red pulp contains predominantly CD68^+^CD163^+^ cells (purple). The mantle zone (M) is primarily occupied by CD68^+^ and Mac387^+^ cells (green and tan, respectively. The marginal zone is a macrophage-rich zone that contains numerous macrophages (CD68^+^, green), perivascular macrophages (CD68^+^CD163^+^; anti-inflammatory, purple), and monocyte-derived macrophage (Mac 387^+^ cells; inflammatory, tan). Macrophages (CD68^+^, green) cells in this region are in closer proximity to the red pulp. (**B**–**D**) With the onset of an SIV infection, splenic macrophages become polarized and express different cell markers during acute, asymptomatic and chronic infection stages. (**B**) Acute SIV infection. In acute infection, perivascular macrophages (CD68^+^CD163^+^, purple; anti-inflammatory) are upregulated within the entire spleen and CD68^+^ cells (green) are substantially reduced. Further, the fetal-derived macrophages (CD68^+^CD163^+^, purple) decline and are replaced by perivascular (CD163) and Mac387^+^ macrophages (blue and tan, respectively) in the red pulp. (**C**) Asymptomatic SIV infection. In this stage, peripheral blood monocytes (CD68^+^, green) enter the spleen and take up residence in germinal centers. During this phase, macrophages partially recover in number within the mantle and marginal zones. (**D**) Chronic/late-stage infection. At this stage of SIV infection, the macrophage populations present in the mantle and marginal zones, as well as in the red pulp observed during the asymptomatic stage, are largely maintained. However, CD68^+^CD163^+^ macrophages (purple) are observed in germinal centers. This may represent a terminal stage mechanism in an attempt to recover the acutely lost CD68^+^ cells (green). Depletion of T-helper (Th) cells after acute disease is indicated by reduced number of Th cells. ca, central arteriole; gc, germinal center; m, mantle; mz, marginal zone; pz, parafollicular zone; PALS, periarteriolar lymphatic sheath; Th, T-helper; SIV, simian immunodeficiency virus; 68, CD68; 163, CD163; 387, Mac387; 68/163, CD68/CD163.

CD68^+^CD163^+^ cells in non-infected (Naïve, Figure 4A) are replaced by CD163^+^ cells that reside in the red pulp evident by acute disease (Figure 4B–D). These CD163^+^ macrophages are a splenic resident macrophage population derived from the same bone marrow precursors as perivascular macrophages and microglia in the brain early in development, and maintain/replace themselves via local cell division in the spleen. CD163^+^ macrophage reside in the red pulp of the spleen, where they become a major viral reservoir for SIV. This is consistent with microglia as an HIV-1 reservoir in the CNS; these cells have a long half-life of years and, unlike monocyte-derived macrophages, maintain the capability of self-renewal and to proliferate in response to brain injury. CD163+ macrophages that populate in the spleen are bone marrow-derived, but their potential to proliferate in response to damage to the spleen or in response to immune activation from cytokines such as TNF-α or IL-1β has not been investigated. There are no reports as to whether SIV or HIV-1 infection is related to local CD163^+^ macrophage proliferation/turnover in the spleen.

Persistent SIV infection and inflammation in the spleen change its structural morphology by profoundly depleting T cells in the periarteriolar lymphatic sheath in the white pulp after acute infection [78]. The CD68^+^, CD163^+^, and CD387^+^ macrophages are highly infected by SIV and reside in the red pulp (Figure 4B–D). Their findings support that macrophages contribute to SIV infection in the spleen by driving morphologic changes and depleting macrophage subsets important for the basic organization of the spleen. Splenic macrophages significantly influence SIV replication and sufficiently maintain viral production in the absence of T cells. These macrophages promote morphologic changes in the spleen through lost macrophage subsets that specifically direct the unique organization of the spleen. Additionally, in infected spleens from SIV and SHIV monkeys, the vascular walls of the arterioles thicken due to perivascular hyalinization, a hallmark of age-related arteriosclerosis [124,125]. Reduced blood flow secondary to arteriosclerosis causes ischemia, infiltration of inflammatory cells, and subsequent necrosis. HIV-1 infection also promotes extramedullary hematopoiesis in the bone marrow, perhaps compensating for inflammation-induced impairment of this function in the splenic red pulp [109,126,127,128,129]. 

Strong evidence supports that SIV or HIV-1 promotes irreparable damage to the splenic mononuclear phagocyte system. Splenic pathogenesis is largely driven by infection and alterations of macrophage and dendritic cell subsets. The assault on macrophages occurs early after infection, and is sustained until end-stage disease. Few HIV-1-infected macrophages undergo apoptosis, consistent with the viruses’ need for long-term residency and reproduction in splenic macrophages. These macrophages have a central role in HIV infection and deregulatory changes in the spleen [108,130,131]. Thus, both viral titers and viral replication are low during viral latency, but are sufficient to sustain chronic infection. However, under conditions of psychosocial stress, SIV or HIV-1 can be reactivated to shift viral latency to active disease [132,133].

In HIV-infected patients with high viral load, immature myeloid pDC that are IFN-α-negative accumulate in the red pulp; other immune cells produce the IFN-α [106]. These macrophages express CD68 and CD163, markers used to identify resident and recently infiltrated macrophages into the spleen, respectively [134,135,136,137]. CD163 is a scavenger receptor and marker for alternatively activated or anti-inflammatory M2 macrophages [78]. Monocytes that express Mac387 migrate into the spleen in SIV infection (Figure 4B–D), and are destructive cells. Mac387 is an antibody with specificity for the L1 antigen (calprotectin) [121]. Newly infiltrated macrophages (Mac387^+^) are distinct morphologically from splenic resident macrophages and differ in their functional capabilities [100,101,105,121,123,135]. These macrophages enter the spleen in response to high viral load early after SIV infection, possibly to mitigate lost CD68^+^ and CD163^+^ macrophages, but once there, they contribute to lymphoid tissue destruction [78].

### 4.3. Activation of Type I Interferon Gene Expression in the SIV-Infected Spleens

SIV infection induces type I interferon expression in the spleen. Early in SIV infection, IFN-β and many of the interferon-stimulated genes significantly increase in the spleen and remain elevated in chronic disease, although at lower levels than in acute disease [78]. IFN-α, which is usually co-produced at comparable levels with IFN-β, is expressed to a lesser extent in early SIV infection. Other genes that are activated by SIV infection to induce type 1 interferons include myxovirus resistance protein 1 (MxA), interferon regulator factor 7 (IRF7), signal transducer and activator of transcription 2 (STAT2), and suppressor of cytokine signaling 3 (SOCS3). Williams et al. [78] suggest lower IL-12 may be responsible for lower IFN-α, as IL-12 promotes IL-10, which inhibits IFN-α [137,138], and IL-10 is elevated in the spleen [78,139,140]. 

IFN-β maintains virus-induced SOCS3 expression in SIV infection. SOC3 promotes M2 polarization [141], consistent with elevated M2-polarizing cytokines, IL-10, indoleamine (IDO)-1, CXCL10, and IL-27 [78]. In contrast, M1 gene expression of Th1 cytokines remains low [78], detrimentally inhibiting cell-mediated immunity. Cultured splenic macrophages polarize toward M1 or M2-inducing cytokines, and return to their resting phenotype when these stimuli are removed [141], supporting their role in establishing SIV infection. This flexibility in M1–M2 conversion is important for returning to homeostasis after clearance of a pathogen.

In SIV infection, different macrophage polarization states characterize acute and chronic inflammation ([1,141] reviewed in [2]). In early disease, the spleen has a type-1 inflammatory microenvironment, but then shifts toward M2 polarization due to a Th1-to-Th2 cytokine shift [78,142,143,144,145]. Both M1 and M2 macrophages become dysfunctional by unique mechanisms in SIV and HIV-1. Recently, Ganor et al. [146] have described an intermediate polarization state referred to as Mi, in which macrophages express markers characteristic of both M1 (IL-1R) and M2 (CD206) phenotypes. Collectively, an anti-inflammatory, IFN-β-driven immune response in the SIV-infected spleen promotes an M1 to M2 macrophage polarization shift, conducive to viral production in the spleen [147,148]. The consequences of an inappropriate, perpetual M2 macrophage phenotype in the SIV spleen are consistent with greater vulnerability to opportunistic infections. Inability to reactivate HIV-1 provirus in the splenic reservoir remains an important barrier to HIV-1 eradication. Lim et al. [149] propose eradication strategies to reactive provirus by cell activation or epigenetic modification. They have shown the PKA activator, dibutyryl-cAMP reactivates HIV-1 by phosphorylating Creb and ATF-1, an effect synergized by an HDAC inhibitor. This line of research may provide a promising strategy for reducing viral reservoir levels in tissues, like the spleen. 

Kelley et al. [32] demonstrate disease-induced sympathetic denervation in the spleen, which may be protective as stressors that activate the SNS promote HIV-1 infection [71,72,73,99,150,151]. Moreover, nerve loss may provide resistance to the shift from M1 to M2 polarization in early disease. Sustained sympathetic nerve loss removes an important immune regulator, particularly one important for turning off an immune response. Increased chronic activation of sympathetic nerves in the spleen promotes M2 polarization, and subsequently a Th2 adaptive immune response, as well as driving profound progressive sympathetic nerve loss in the spleen [151]. In early disease, the presence of sympathetic nerves in the spleen may favor M2 macrophage polarization, whereas their subsequent loss may suppress M2 polarization and a Th2 response, and support a steady-state viral reservoir. Moreover, M2 polarization promotes a reduced capacity to support productive CCR5-dependent HIV-1 infection in HIV-1 latency [144] and a steady-state viral reservoir [152]. A perpetual M2 macrophage phenotype also comes at the expense of the spleen’s ability to develop an appropriate inflammatory response to pathogens that enter the spleen with circulating blood, and may contribute to greater vulnerability to opportunistic infections in HIV-1.

These findings underscore the importance of the spleen in SIV and HIV infection, and the vulnerability of HIV patients to opportunistic infections as the normal immune functions of this organ are compromised by HIV infection. Since the spleen inserts itself in the systemic blood circulation, monocytes enter and exit the cardiovascular system at this site [153,154]. Therefore, infected monocytes leaving the spleen can promote viral seeding of other organ systems, which is a challenge for developing HIV-1 curative treatments. Therapeutic strategies must also focus on restoring normal phenotypic macrophage populations and their functions, and limit macrophage infection in the spleen.

## 5. Functional Consequences of SIV-Induced Pathology in the Spleen

SIV induces fluctuations in cellular and humoral immunity through end-stage disease. Differential regulation of macrophage expression of CD68 and CD163 by SIV infection markedly impairs splenic function and integrity. SIV induces the downregulation of functional markers in splenic macrophages that impairs their activation and their ability to filter the blood of blood-borne pathogens. Impaired activation of splenic macrophages in the white pulp promotes splenic pathology, and compromises the ability of the immune system to respond appropriately to opportunistic infections [115]. This is consistent with HIV-1 patients vulnerable to extremely poor outcomes for what are normally non-lethal types of infections by compromising cellular immune functions. This includes failure to generate antigen-specific immune responses that protects against blood-borne infections from viruses, bacteria and fungus. In the red pulp, apoptosis of embryonic originating CD68^+^ macrophages has profound implications for splenic function. Once these cells are lost, there is limited potential to restore them in adults. The shift in CD68^−^ and CD68^+^CD163^−^ macrophages to a CD163 phenotype (Figure 4B) has functional implications in their ability to defend against opportunistic infections, since these cells serve as antigen-presenting cells that are required for mounting effective adaptive immune responses. These cells are important for clearing damaged erythrocytes, and scavenging iron from dead red blood cells. Newly disseminated inflammatory Mac387 macrophages occur transiently in the lymph nodes of SIV-infected macaques, where they promote lymphoid tissue destruction, viremia, and pathology in ganglia that innervate lymphoid tissue [155,156]. During early symptomatic disease, their entry into the spleen significantly drops, possibly from preferential cell death.

Peripheral blood monocytes are abnormally recruited to the spleen after HIV-1 infection, particularly by the chemokine, CCL8, a ligand that binds to CCR5. CCL8 is regulated temporally by monocyte chemokines and chemokine receptors. CCL8 also inhibits replication of HIV-1. While CD68, CD163, and Mac387 macrophages are all susceptible to SIV infection, Mac387 macrophages are the least susceptible to infection, possibly because these cells are relatively immature and express less CCR5 [157]. CD68 macrophages are more permissive to SIV infection than Mac387 cells, but their infection is temporally regulated. CD163 cells are distinct from the other macrophage subsets as they are highly susceptible to SIV and incredibly resilient to high viral load, consistent with being a preferred residence for SIV. 

T cells are highly infected during acute disease, but the number of SIV-infected T cells significantly declines after acute infection and remains low thereafter. Preferentially infected T cells are confined to follicles in the white pulp. T cells are minimally infected in comparison with CD163^+^ macrophages during chronic disease, after their significant depletion from acute infection, a time when viral load is high. In contrast, infected macrophages predominate in the red pulp. SIV infection of myeloid cells in spleen occurs independently of T cells, and infected red pulp macrophages contribute greatly to viral burden in the spleen. In addition, red pulp macrophages are a source of prolonged viral production, because they escape SIV-cytotoxic T lymphocyte killing, even though they share residency in the red pulp [158,159].

## 6. HIV-1 Infection and Consequences for Sympathetic Innervation of the Spleen

A common long-term side effect of HIV is peripheral neuropathy, and remains one of the most common long-term side effects. Causes of peripheral neuropathy in AIDS patients are attributed to HIV infection of non-neuronal cells within the ganglia, particularly infiltrated macrophages. Peripheral neuropathy is observed in the presence and absence of antiretroviral therapy. The inflammatory environment in the spleen of HIV infected individuals compromises the ability of the nervous system to regulate innate and adaptive immunity. Early in disease, HIV-1-infected macrophage and/or its viral proteins may invade the superior celiac–mesenteric ganglion, as has been reported by Esiri et al. [160] in other autonomic ganglia (i.e., inferior cervical and sympathetic chain ganglia). The superior celiac–mesenteric is the ganglionic complex that supplies the spleen with sympathetic nerves. Our report of sympathetic innervation loss in the spleen in mice with MAIDS supports damage to these neurons. Early HIV infection induces a cytokine storm that supports early viral replication, neurotoxic viral protein production/secretion, and later a chronic inflammatory microenvironment that promotes neuronal dysfunction [160,161,162,163,164]. 

Under proinflammatory conditions, infiltration of macrophages and T cells into the superior mesenteric–celiac ganglia may play a role, at least in part, in the profound loss of sympathetic nerves in the spleen [160]. Inflammation in these ganglia may come at a cost, as inflammation can promote apoptosis-induced neuronal cell loss [160,161,162]. During HIV-1 exposure, genes are upregulated that strongly support interferon-induced responses and antigen presentation in cultured neuron-like cells [161]. In response to HIV exposure, these cultured neurons upregulate their expression apolipoprotein E, which influences the anti-HIV innate immune response in these cells [162]. This research indicates that neuroepithelial progenitors and their differentiated progeny recognize HIV-1 and mount an innate immune response and the rigor of this response is influenced by apolipoprotein E, which has protectives effects in sympathetic neurons against apoptosis.

Damage of sympathetic neurons that innervate the spleen is an early systemic event in MAIDS [32], and is consistent with early inflammation in HIV-1 infection [160,161,162,163] and active viral replication in non-neuronal cells in the ganglia during acute infection [164]. Infected macrophages may enter the superior mesenteric–celiac ganglia during acute infection, as well the endothelial cells of the microvasculature that distribute blood to these cells [160]. Local macrophages may provide early partial viral clearance in asymptomatic infection, and viral load may decline in response to the innate antiviral response generated by macrophages. HIV-1 infection drives chronic inflammation and cell processes that are degenerative [165,166] in the process of propagating local innate immunity. Local resident and infiltrated macrophages secrete inflammatory chemokines, cytokines, and oxidative free radicals that incite oxidative injury. They also ramp-up neurotoxic excitatory amino acids, such as glutamate and aspartate, that activate innate immunity and are neurotoxic. Similarly, viral proteins, such as Tat, p41 envelope protein, and gp120, are neurotoxic and secreted by infected T cells and macrophages. An inflammatory response ensues and provides a microenvironment that is permissive for neuronal apoptosis and/or axonal injury in HIV-1 infection [165,166].

**Figure 5:**
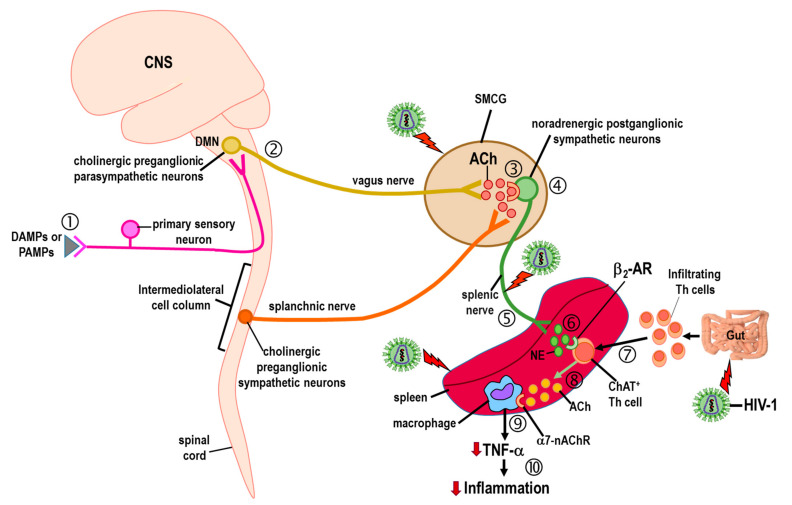
The Cholinergic Anti-inflammatory Pathway and Anti-inflammatory Response and HIV-1. The cholinergic anti-inflammatory pathway is an efferent two-neuron circuit, whereby the parasympathetic neurons in the dorsal motor nucleus (DMN) of the vagus nerve (gold neuron) inhibits proinflammatory cytokine release by cells of the immune system via their innervation of sympathetic neurons (green) in the superior mesenteric-celiac ganglion (SMCG). In this diagram, the DMN of the vagus receives and integrates information from vagal afferent sensory neurons (pink) that sense damage-associated and pathogen molecular patterns (DAMPs and PAMPs, respectively; gray triangle, (1)). The efferent arm of the vagal reflex arc arises from the dorsal motor nucleus (DMN) of the vagus (2) and terminates onto postganglionic sympathetic neurons in the superior mesenteric-celiac ganglion (SMCG, (3)). These vagal nerves modulate the sympathetic nerve activity (4) to regulate inflammation. Preganglionic cholinergic sympathetic neurons (orange) in the intermediolateral cell column signal noradrenergic postganglionic neurons (green). Axons from sympathetic SMCG neurons (green) innervate the spleen via the splenic nerve (5) where they form neuroeffector junctions with choline acetyltranserase (ChAT)-positive (ChAT^+^) Th cells (6) that have migrated from the gut into the spleen (7). The neurotransmitter, norepinephrine (NE, green circles) release from sympathetic nerve terminals binds to β_2_-adrenergic receptors (β_2_-AR) expressed on the cell surface of gut-derived ChAT^+^ positive Th cells. NE binding with β_2_-AR stimulates gut-immigrated Th cell secretion of acetylcholine (ACh, orange circles; (8)), which binds to α_7_-nicotinic acetylcholine receptors (α_7_-nAChRs) expressed on cell surface of macrophages (9) to inhibit their tumor necrosis factor (TNF)-α secretion (10). HIV-1 and/or its viral proteins can disrupt the cholinergic anti-inflammatory response, tampering with a major pathway that controls inflammation at multiple sites (virus-red lightning bolts; see ref. [160]). In this way, HIV-1 infection promotes chronic inflammation by inhibition of the cholinergic anti-inflammatory pathway. The orange neuron represents preganglionic cholinergic sympathetic neurons in intermediolateral cell column that also converge onto postganglionic noradrenergic SMCG neurons via the splanchnic nerve that innervates in the spleen. CNS, central nervous system; HIV-1, human immunodeficiency virus-1. Black arrows, directional; red arrows, inhibition.

Loss of sympathetic innervation of the spleen has implications for the vagally mediated cholinergic anti-inflammatory response in HIV infection. Vagal dysfunction is reported in HIV-1, with some support for the vagus nerve as a mediator of HIV-1-induced bacterial overgrowth in the small intestines and elevated proinflammatory IL-6 [167]. The underlying mechanism(s) responsible for this nerve–gut interaction remain a mystery, but may be related to gastrointestinal changes in motility and/or its microbiome. Any influence of HIV-1-mediated dysfunction in the spleen by changes in the signaling of the vagus nerve must be indirect, because the vagus nerve does not innervate the spleen (Figure 5). However, a cholinergic anti-inflammatory pathway has been described in which sympathetic nerves that innervate the spleen regulate the secretion of acetylcholine from splenic macrophages. The naming of this pathway, the cholinergic anti-inflammatory pathway, breaks conventional naming of pathways, as historically, neurotransmitters that describe neural pathways are typically secreted by the last group of neurons innervating the effector tissues in the pathway, not by the mediators secreted by the cells they regulate. 

In the cholinergic anti-inflammatory pathway (Figure 5), cholinergic nerves from the dorsal motor nucleus of the vagus innervate sympathetic neurons in the superior-mesenteric celiac ganglion. These sympathetic noradrenergic neurons, in turn, innervate the spleen [23]. Under systemic inflammatory conditions, the activity of splenic sympathetic nerves increases, causing greater release of norepinephrine from their nerve terminals. Released norepinephrine then binds to ARs expressed in cholinergic Th cells that are deployed from the gut into the spleen during systemic inflammation. Adrenergic stimulation of gut-immigrant Th cells secretes acetylcholine that binds to α7-nicotinic cholinergic receptors (α7-nAChRs) expressed on the cell surface of splenic macrophages. Engagement of these receptors suppresses macrophage secretion of TNF-α, suppressing inflammation. Therefore, virally induced sympathectomy in the spleen is expected to disrupt the function of the cholinergic anti-inflammatory pathway. 

## 7. HIV-1 and Inflammation in Autonomic Ganglia

Autonomic ganglia are infected early after HIV-1 infection. Viruses can freely enter the ganglia, because there is no barrier to entry [168]. Unlike the CNS whose blood–brain barrier prevents viral entry during early disease, blood vessels in autonomic ganglia no such barrier, allowing the infiltration of healthy and infected immune cells to infiltrate sympathetic ganglia, with infected immune cells contributing to the spread of HIV-1. Locally, astrocyte-like satellite glial cells and pericytes that support the health of sympathetic neurons can be infected with HIV-1 (reviewed by [169]).

In autonomic ganglia, HIV-1 induces host cells to produce and secrete viral proteins that mediate inflammatory and toxic effects in sympathetic neurons [170]. Additionally, inflammation of sympathetic ganglia may contribute to heightened sympathetic nerve activity in the spleen, with secondary consequences for the autodestruction of their nerve endings. Tat is secreted by infected, immune-activated satellite glial cells [170]. These cells may amplify inflammatory signals from HIV-1-infected pericytes through the secretion of inflammatory, interferon-inducible factors. HIV-1 Tat protein can suppress nerve growth factor, which may contribute to sympathetic nerve loss in the spleen [170]. These microenvironmental conditions exert stress on sympathetic neurons that can potentiate neuronal apoptosis [171]. In the initial stages of HIV-1 invasion, autonomic ganglia are not isolated from the systemic immune response. Thus, the immune response within the ganglia is relegated to innate mechanisms generated by specialized satellite ganglion cells. Under normal conditions, innate immune defense within neural tissue is, by necessity, immediate, controlled and, importantly, neuroprotective. However, this is not the case for HIV-1 infection, where the cells in charge of immune defense are infected and hijacked by the virus. As a result, innate immune responses in sympathetic ganglia in HIV-infected individuals propagate a chronic inflammatory microenvironment. HIV-1 infection induces the activation of infected innate microglia-like immune cells that damages bystander neurons that they are normally tasked to protect.

Because neurons lack receptors for CD4 and CXCR5-binding chemokines that are required for viral entry into cells, they are not infected by HIV-1. TLRs expressed in neurons support at least a minor role in host defense via several mechanisms. Human neurons can recognize dsRNA by the TLR3 they express [172]. Their activation in response to a viral infection elicits a strong inflammatory response that includes the release of TNF-α, IL-6, CCL-5, CXCL-10, 2–5′-oligoadenylate synthetase (2′5′ OAS), and interferon-β [172]. Additionally, complement can potentiate the immune sensing of HIV-1 and early Type I interferon responses [173]. Proinflammatory cytokines are induced in neurons after MHC-I stimulation by proinflammatory molecules, including cytokines [174,175], and importantly viral infections [176], with the potential for antigen presentation to infiltrating CD8 cells in later stages of HIV infection [177]. These findings support that these sympathetic neurons can generate some immune mechanisms to defend against HIV-1, and these mechanism can recruit cells of the immune system. Instead of being active participants in HIV pathogenesis, sympathetic neurons are injured due to a persistent inflammatory microenvironment, and a target of HIV-1 secreted proteins.

Collectively, existing findings support a two-front assault on sympathetic neurons that supply the spleen. This may explain the severity of sympathetic nerve damage that Kelley et al. [29] report in the spleens of mice with MAIDS. The profound loss of sympathetic nerves in spleens from mice with MAIDS was an acute event. Given this two-front assault (targeting the spleen and the ganglion neurons), it seems unlikely that these nerves regenerate considering the magnitude of multipronged assault on these neurons. However, this hypothesis will need to be verified, especially given the resiliency of these nerves to other types of immune-induced insults. Still, the severe loss of sympathetic nerves (and likely neurons) is much more profound than what we have previously observed for mice treated with the neurotoxin, 6-hydroxydopamine [178]. It remains to be determined whether the nerves have retracted from the hostile inflammatory microenvironment of the MAIDS spleen or whether the dual attack of HIV-1 on the sympathetic cell bodies also induces apoptotic cell death. Additionally, the role of HIV-1 regulatory proteins, such as Tat, Vpu, Nef, Vpr, and Rev, remains understudied, but there is growing evidence that they will play important roles in nerve structural and functional changes induced by HIV-1 infection [179,180,181].

**Figure 6:**
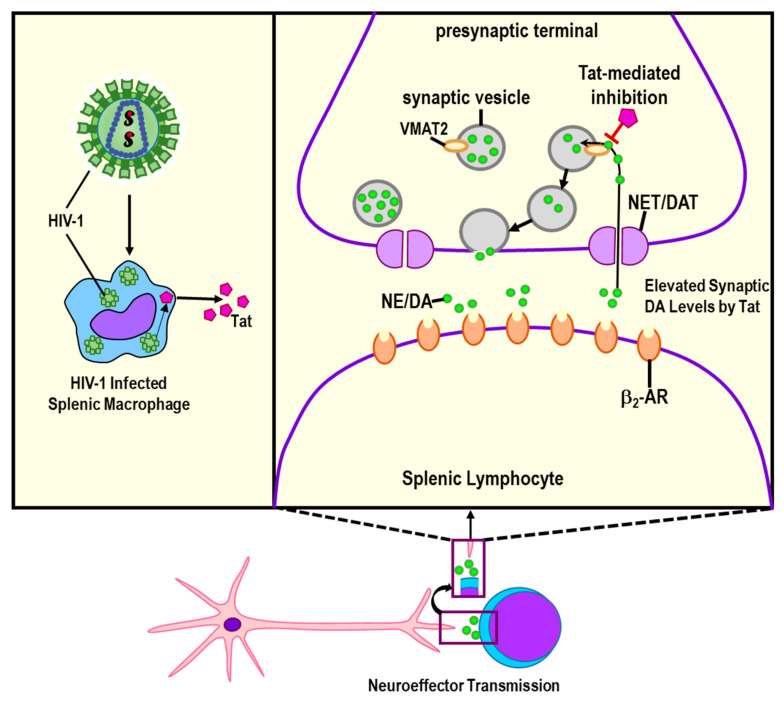
Sympathetic neurons in the superior–celiac ganglia innervate cells of the immune system in the spleen. A representative neuron (left side, pink) innervating a splenocyte (right side) is illustrated at the bottom of this figure, and the neuroeffector synapse with released neurotransmitter (green circles) is shown inside the black box. The image inside and the box is turned 90 degrees (indicated by black arrow) and magnified (indicated by the dashed lines, upper right panel) to illustrate the neuroeffector synapse with a splenic lymphocyte. Tat and other viral proteins (e.g., gp120, not shown) are secreted from infected splenic macrophages (left panel). Secreted Tat is taken up by sympathetic neurons by dopamine and norepinephrine transporters (DAT and NET, respectively) (right panel). Tat binds to vesicular monoamine transporter 2 (VMAT2), preventing the uptake of dopamine (DA) and norepinephrine (NE) into synaptic vesicles (right panel). Thus, inhibition of VMAT2 blocks an important step in the loading of these monoamine neurotransmitters into synaptic vesicles in sympathetic nerves. Unsequestered NE and DA are neurotoxic to sympathetic nerves, promoting the activation of p53 family members, and may be contributory to HIV-1-induced sympathectomy. AR, adrenergic receptor; HIV-1, human immunodeficiency virus-1.

The effects of Tat on sympathetic nerve integrity have been the most studied. There are existing data to support that Tat, as well as gp120 and to a lesser extent Nef, play important roles in the profound loss of sympathetic nerves in the spleen. It is well documented that Tat binds to vesicular monoamine transporter 2 (VMAT2) (Figure 6), an integral membrane protein in sympathetic neurons. VMAT2 transports monoamines, particularly sympathetic neurotransmitters including dopamine and NE, from the cytosol into synaptic vesicles [179]. Incorporation of the catecholamines, NE, dopamine and epinephrine into synaptic vesicles is required to protect sympathetic nerves from the oxidative stress-induced nerve damage that these neurotransmitters cause if they are not packaged into vesicles. Once packaged, the vesicles are transported down the sympathetic axons for release of catecholamines (e.g., dopamine and NE) at varicose synaptic sites along the axon. After release, NE interacts with α- and β-ARs that reside on the cell surface of all classes of immune cells [68]. A more extensive review of how the SNS regulates innate immune function is reported in Bellinger and Lorton [68]. An important function of the sympathetic regulation of immunity in the spleen and other secondary immune organs is their anti-inflammatory drive and their role in resolving immune responses to restoring homeostasis.

In this review, we discuss, clarify, summarize and integrate the available literature regarding infection in the spleen by retroviruses that induce AIDS and animal models for AIDS, restricting our focus to innate immunity. Complex interactions occur between the viruses and the host’s immune system that challenge the host’s immune system. Moreover, these viruses do not stop with the immune system. Either by direct attack or by secondary consequences of acute and chronic inflammation, HIV-1 and other retroviruses that cause AIDS in animal models damage sympathetic neurons that provide a regulatory influence over innate and adaptive immunity. Here, we focused our review specifically on innate immunity in the spleen, a major site of HIV-1 infection and an immune organ that is well regulated by the sympathetic nervous system. Both the sympathetic and vagal pathways are important for modulating innate and adaptive immune response with the goal of facilitating pathogen eradication and minimizing damage from inflammation. Available research supports marked damage of sympathetic pathways that supply the spleen during acute infection and are required for parasympathetic regulation of immune function. Loss of sympathetic nerves, and thus, sympathetic regulation of inflammatory cytokines is expected to promote chronic inflammation. The loss of sympathetic innervation in the spleen may enhance IFN-β secretion by myeloid suppressor cells that promotes HIV-1 replication. Because the spleen is positioned within the circulatory system, HIV-1-induced inflammation is systemic. Both sympathetic and sensory nerves are vulnerable to neuropathy from chronic inflammation. Additionally, the inhibition of IFN-β synthesis in infected myeloid cells by norepinephrine early in disease may facilitate viral replication. However, the early loss of these nerves in the spleen is expected to increase IFN-β synthesis and consequently promote viral replication. More research is needed to flesh out the timing of these events to gain a better understanding of neural–immune interactions in AIDS. 

Despite the time that has passed since our initial studies, many gaps in our knowledge of sympathetic-immune regulation in the spleen and the pathogenic consequence of HIV-I infection in the spleen remain unanswered. In summary, sympathetic modulation of innate immunity remains understudied, and poorly understood, particularly with respect to antiviral immunity [68,73]. Adrenergic receptor stimulation of innate immune cells plays roles in antigen detection and processing/presentation in APCs that interface downstream with intracellular pathways activated by pattern recognition receptors. Diversity in the responses by β-AR activation may result from the multiple isoforms of adenylate cyclase and PKA [149]. This diversity also is due to promiscuity in receptor coupling to either Gs or Gi, or uncoupling from adenylate cyclase to other signaling pathways. Our current understanding of how and where sympathetically activated intracellular pathways in immune cells converge onto those activated by immune stimuli remains sparse. However, with further research, we can begin to make reasonable predictions about local and systemic outcomes in vivo under normal and disease-related conditions. Similarly, the spleen is an understudied organ in the context of HIV-1. Gaps in our knowledge include whether treatment with a β_2_-adrenergic blocker would be protective against HIV-1 induced inflammation, or how it protects the blood–brain barrier and reduces systemic pathology induced by HIV-1 Nef protein [181]. Obtaining this information should help bridge the gap in our understanding, which has the potential to harness neural-immune-related strategies/therapies to protect nerve integrity, limit the hostile takeover of splenic macrophages and Th and memory cells, and restore antiviral immunity. This knowledge will facilitate the development of novel pharmaceuticals tools tailored to treat autonomic dysfunction in HIV-1 infection.
